# The impact of socio‐economic deprivation on the long‐term survival of people with diabetes and acute myocardial infarction: A nationwide cohort study

**DOI:** 10.1111/dme.70111

**Published:** 2025-07-28

**Authors:** Nicholas Weight, Andrew Cole, Muhammad Rashid, Kamlesh Khunti, Shivani Misra, Evangelos Kontopantelis, Thomas A. Shepherd, Martin K. Rutter, Mamas A. Mamas

**Affiliations:** ^1^ Keele Cardiovascular Research Group, Centre for Prognosis Research, Institute for Primary Care and Health Sciences Keele University Keele UK; ^2^ Department of Cardiovascular Sciences University of Leicester Leicester UK; ^3^ National Institute for Health Research (NIHR) Leicester Cardiovascular Biomedical Research Unit Glenfield Hospital Leicester UK; ^4^ Diabetes Research Centre University of Leicester Leicester UK; ^5^ Department of Metabolism, Digestion & Reproduction Imperial College London London UK; ^6^ Division of Informatics, Imaging and Data Sciences University of Manchester UK; ^7^ School of Medicine Keele University Keele UK; ^8^ Diabetes, Endocrinology and Metabolism Centre Manchester University NHS Foundation Trust, NIHR Manchester Biomedical Research Centre Manchester UK; ^9^ Division of Diabetes, Endocrinology and Gastroenterology, School of Medical Sciences, Faculty of Biology, Medicine and Health University of Manchester Manchester UK; ^10^ National Institute for Health and Care Research (NIHR) Birmingham Biomedical Research Centre Birmingham UK

**Keywords:** acute myocardial infarction, diabetes mellitus, long‐term mortality, socio‐economic deprivation

## Abstract

**Background:**

People from areas of socio‐economic deprivation have poorer outcomes following acute myocardial infarction (AMI). How deprivation influences the survival of people with diabetes mellitus (DM) post‐AMI is not well described.

**Methods:**

Using the Myocardial Ischaemia National Audit Project (MINAP) registry, with Office for National Statistics (ONS) mortality recording, 729,722 patients from England and Wales between 2005 and 2019 were included, 152,867 with DM and followed up to 31 July 2021. Patients were stratified into quintiles using the Index of Multiple Deprivation (IMD) score (Q1—most‐deprived, Q5—least deprived), Cox regression models were fitted and adjusted mortality risk estimates were compared by IMD quintile and DM status.

**Results:**

Thirty‐day mortality risk between Q1 (most deprived) and Q5 (least deprived) was similar for patients with DM, but in patients without DM, risk was higher in the most deprived group (aHR: 1.05 (1.01–1.09), *p* < 0.001). Risk of 1‐year (aHR: 1.05 (1.01–1.10), *p* < 0.001), 5‐year (aHR: 1.14 (1.11–1.17), *p* < 0.001) and overall mortality (aHR: 1.14 (1.12–1.17), *p* < 0.001) was higher in Q1 compared to Q5 for patients with DM, but this increase was smaller than in patients without DM at 1 year (aHR: 1.12 (1.09–1.14), *p* < 0.001), 5 years (aHR: 1.18 (1.16–1.20), *p* < 0.001) and overall (aHR: 1.22 (1.20–1.23), *p* < 0.001). Adjusted 1‐year mortality was higher in patients with DM than those without DM regardless of IMD quintile (e.g. DM vs. non‐DM: Q1: 18.8% vs. 16.1%, Q5: 17.8% vs. 14.6%).

**Conclusion:**

Patients with DM from socio‐economically deprived regions have a higher risk of mortality at 1 year, 5 years and overall, compared to least deprived patients with DM following AMI. However, the inequality gap was larger in the non‐diabetic population, suggesting that current approaches to management in people with DM may mitigate some of the effect of deprivation on outcomes.

## Graphical abstract

Graphical Abstract summarising key study findings.

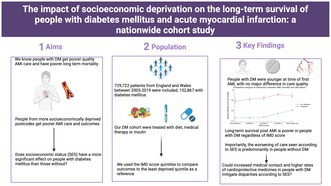

**What's new?**

**What is currently known about this topic?**

- Acute myocardial infarction (AMI) quality of care, in‐hospital and long‐term clinical outcomes are known to be poorer for patients with diabetes mellitus (DM).
- Similar disparities also exist for patients from the most socio‐economically deprived areas, with poorer in‐hospital quality of care and in‐hospital outcomes recently demonstrated.

**What is the key research question?**

- Does poorer socio‐economic status affect patients with DM more so than those without?

**What is new?**

- The impact of poorer socio‐economic status on long‐term survival post AMI was less significant in patients with DM compared to those without.
- Quality of care did not vary according to socio‐economic status in patients with DM.
- We suggest that more regular medical contact and higher rates of cardioprotective medicines such as statins are contributing to this effect.

**How might this study influence clinical practice?**

- Patients with poorer socio‐economic status without diabetes mellitus are at particular risk of socio‐economic disparities in AMI care.
- Our study reiterates the importance of starting cardioprotective medicines in patients with DM early in their disease process to improve long‐term cardiovascular outcomes.

## 1 INTRODUCTION

Acute myocardial infarction (AMI) remains a major cause of mortality and morbidity in patients with diabetes mellitus (DM).^1^ This is especially important given the growing burden of DM in an increasingly older and multimorbid AMI population, projected to increase substantially over the following decade.^2^, ^3^

Socio‐economic status (SES) is a well‐established cardiovascular risk factor, especially income level, educational status, employment status and neighbourhood factors.^4^, ^5^ Compared to people from the most deprived areas, those least deprived tend to receive faster identification of modifiable risk factors, higher‐quality guideline‐directed management of cardiovascular risk factors such as hypertension and DM and better in‐hospital care post AMI.^6^, ^7^

Importantly, patients with poorer SES have been shown to be at elevated risk of developing DM; this is multifactorial, including ethnicity and modifiable risk factors such as obesity, alcohol intake, smoking^8^ and educational attainment.^9^ Data from several studies have shown higher risks for cardiovascular mortality in people with T2DM and lower SES,^10^, ^11^, ^12^, ^13^ but relationships between SES and mortality risks specifically in AMI patients are unknown. It is also unclear whether deprivation is related to adverse mortality risks in people with and without DM. It is possible that the adverse CVD impacts of low SES might be offset by the structured education and regular monitoring/intervention for modifiable cardiovascular risk factors routinely offered to all people with diabetes.

Using a comprehensive registry of AMI admissions from England and Wales from 2005 to 2019, linked to long‐term mortality data, we aimed to assess whether DM status influences mortality risk differently across strata of SES, as defined by the Index of Multiple Deprivation. We hypothesised that low SES would be less strongly related to higher mortality risks in people with AMI and DM compared to those with AMI without DM.

## 2 METHODS

### 2.1 Study design

We used the Myocardial Ischaemia National Audit Project (MINAP), a prospective national registry of patients admitted to hospitals in the United Kingdom with an ACS.^14^ The MINAP dataset comprises 130 variables, including baseline demographics and clinical characteristics, comorbidities, management strategies, pharmacotherapy, in‐hospital clinical outcomes and discharge diagnosis.^15^ Data are submitted by hospital clinical staff, and approximately 90,000 pseudonymised records annually are uploaded to the National Institute for Cardiovascular Outcomes Research (NICOR). In‐hospital mortality is recorded in MINAP, but for longer‐term mortality analysis, we used data from the Office for National Statistics (ONS), which is the UK's independent producer of official statistics, regularly collecting data regarding every death registered in the United Kingdom, coding deaths according to the international classification of diseases (ICD‐10) and cause of death from the Medical Certificate of Cause of Death (MCCD).

### 2.2 Study population

**FIGURE 1:**
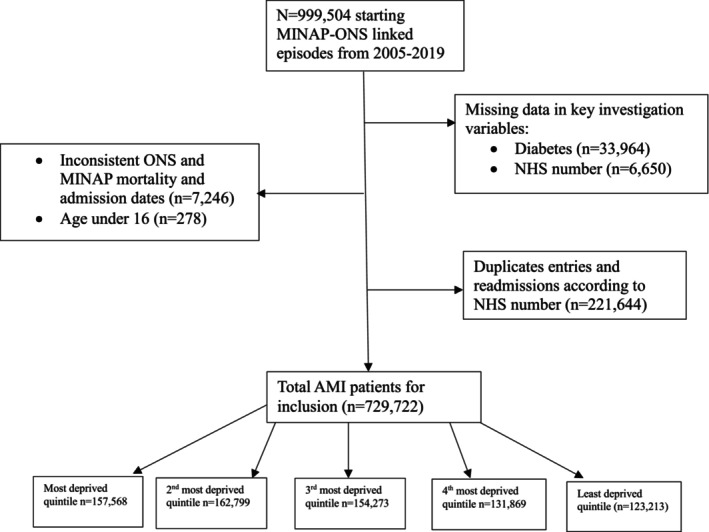
STOBE diagram detailing study inclusion and exclusion criteria.

We included patients admitted with a diagnosis of AMI (STEMI and NSTEMI) in any of the 230 participating hospitals in England and Wales between January 2005 to March 2019. The discharge diagnosis of AMI was determined by local clinicians according to presenting history, clinical examination and the results of in‐patient investigations in keeping with the consensus document of the Joint European Society of Cardiology (ESC) and American College of Cardiology (ACC).^16^ Patients were excluded if they had missing data in key variables for investigation, Index of Multiple Deprivation score, DM status, inpatient mortality, MACE or inconsistent mortality dates (See Figure 1 for full list of exclusions and relevant numbers). The index admission with AMI over the study period is included for patients; duplicate NHS numbers indicating readmissions of patients already included in the study were all excluded from the analysis. Patients were organised into quintiles according to the pre‐existing cut‐offs for the Index of Multiple Deprivation score.^17^

### 2.3 Outcomes

#### 2.3.1 Primary

Primary outcomes of interest were risks for 1‐year and 5‐year all‐cause mortality. We also assessed risk for ‘overall’ all‐cause mortality using all available follow‐up data. Mortality dates were calculated from the date of admission with AMI (as recorded in MINAP) and the date of death as recorded by the ONS. Mortality follow‐up was available up to 31 July 2021 for all included patients in the dataset via a single data download from the ONS.

#### 2.3.2 Secondary

Secondary outcomes of interest for AMI patients were the Opportunity‐Based Quality Indicators (OBQI), which consist of prescriptions for aspirin, P2Y12 inhibitors, beta‐blockers, statins, ACE inhibitors/ARBs and whether referral to cardiac rehabilitation was made. Additionally, for NSTEMI patients, the ESC ACVC Quality indicators for NSTEMI, including whether patients underwent invasive coronary angiography (ICA) within 72 h, whether patients received dual antiplatelet therapy at discharge, and whether LV function was measured during admission were assessed.

### 2.4 Statistical analysis

Demographics, clinical characteristics and crude adverse outcomes of patients by quintile of deprivation were compared using Pearson's chi‐square test for categorical variables. Continuous variables were compared using Student's t‐test, if normally distributed, and using the Wilcoxon rank sum test if not. The normality of distribution was assessed using the Shapiro–Wilk test. Continuous variables are presented as medians and interquartile ranges (IQR), means for non‐normally distributed data and categorical variables by proportions. For ease of presentation, Quintiles 1 and 5 are presented in Tables 1 and 2, with full descriptive Tables for all quintiles included as Tables S1 and S2. Multiple imputations with chained equations (MICE) were used to impute values for variables with missing data. MICE is the best practice when dealing with missing data and can provide unbiased estimates even with high levels of missingness, and some protection when data are missing not at random.^18^ Cox regression models were fitted and applied to 10 imputed datasets, with estimates combined using Rubin's rules.^19^ Models were adjusted for age, sex, ethnicity (classified according to the MINAP categories; White, Black, Asian, Mixed and Other), year of admission, hospital region, admission heart rate, admission systolic blood pressure, co‐morbid conditions (hypertension, history of asthma or chronic obstructive pulmonary disease (COPD), history of cerebrovascular accident (CVA) or peripheral vascular disease (PVD), hypercholesterolaemia, family history of coronary artery disease, smoking history, previous AMI, history of angina, history of previous PCI and previous CABG), medication strategy (aspirin, P2Y12 inhibitor, LMWH, fondaparinux, warfarin, unfractionated heparin (UFH), glycoprotein B3A, ACE inhibitor/ARB, statin, beta‐blocker), cardiac arrest, left ventricular (LV) systolic function, Killip classification and ischaemic ECG changes, invasive coronary angiography and revascularisation by percutaneous coronary intervention (PCI) or coronary artery bypass grafts surgery (CABG). Hazard ratios shown are from comparison of patients from the least deprived quintile according to IMD score. Separate Cox models were run to obtain estimates for 30‐day, 1‐year, 5‐year and ‘overall’ (over the full available follow‐up period) mortality. Kaplan–Meier curves were plotted to demonstrate unadjusted survival, while adjusted survival curves were obtained from the fitted models. The proportional hazards assumption was evaluated in all models by assessing Kaplan–Meier charts and log–log plots.

**TABLE 1:** Demographic comparison for patients admitted with AMI according to socio‐economic status, stratified by presence of diabetes mellitus.

Variables	Quintile 1 (most deprived)		Quintile 5 (least deprived)	
	Diabetes mellitus (*n* = 38,406)	No DM (*n* = 119,165)	DM (*n* = 21,132)	No DM (*n* = 102,088)
Age, years, median (IQR)	69.8 (59.7–78.2)	65.9 (54.7–77.7)	75.2 (66.3–82.2)	72.2 (61.3–81.9)
Woman (%)	14,386/38,406 (37)	41,201/119,165 (35)	6824/21,132 (32)	33,274/102,088 (33)
BMI, median [IQR]	29.2 (25.6–33.5)	26.9 (23.7–30.5)	28.0 (24.8–31.7)	26.3 (23.6–29.4)
Ethnicity—White (%)	16,456/21,193 (78)	54,273/59,510 (91)	10,555/11,368 (93)	49,795/51,137 (97)
Ethnicity—Asian (%)	4092/21,193 (19)	4263/59,510 (7)	737/11,368 (6)	1116/51,137 (2)
Ethnicity—Black (%)	578/21,293 (3)	806/59,510 (1)	54/11,368 (0)	153/51,137 (0)
Ethnicity—Mixed (%)	67/21,293 (0)	168/59,510 (0)	22/11,368 (0)	73/51,137 (0)
Basal crepitations (%)	3157/19,021 (17)	6317/52,950 (12)	1731/10,610 (17)	5149/47,145 (11)
Pulmonary oedema (%)	1582/19,021 (8)	2246/52,950 (4)	840/10,610 (8)	1913/47,145 (4)
Cardiogenic shock (%)	339/19,021 (2)	810/52,950 (2)	164/10,610 (2)	680/47,145 (1)
ECG ST changes (%)	31,267/36,996 (85)	100,300/115,222 (87)	17,253/20,319 (85)	85,469/98,371 (87)
Previous smoker (%)	12,554/36,398 (34)	31,777/114,461 (28)	7993/19,631 (41)	33,574/96,476 (35)
Current smoker (%)	10,178/36,398 (28)	50,580/114,461 (44)	2523/19,631 (13)	17,974/96,476 (19)
CCF (%)	3219/34,817 (9)	4854/108,109 (4)	1722/19,860 (9)	4029/96,309 (4)
Hypercholesterolaemia (%)	16,736/34,829 (48)	30,460/107,750 (28)	8818/19,788 (45)	27,294/95,713 (29)
Cerebrovascular disease (%)	4367/34,837 (13)	8242/108,318 (8)	2345/19,913 (12)	6733/96,342 (7)
History of angina (%)	12,687/35,081 (36)	22,565/108,744 (21)	6308/19,990 (32)	18,109/96,875 (19)
Peripheral vascular disease (%)	2874/34,567 (8)	4245/107,462 (4)	1514/19,715 (8)	2821/95,306 (3)
Chronic renal failure (%)	4185/34,786 (12)	4319/108,016 (4)	2393/19,855 (12)	4104/96,259 (4)
Hypertension (%)	24,151/35,563 (68)	47,078/109,803 (43)	13,775/20,248 (69)	45,205/97,647 (46)
Asthma/COPD (%)	7477/34,782 (22)	20,502/107,867 (19)	2604/19,785 (13)	11,241/95,492 (12)
Family history of CAD (%)	8275/27,954 (30)	30,686/90,237 (34)	4288/15,958 (27)	25,014/80,583 (30)
Previous AMI (%)	10,365/35,289 (29)	17,642/109,503 (16)	5134/20,144 (25)	13,758/97,510 (14)
Previous PCI (%)	4206/34,814 (12)	6336/108,233 (6)	2329/19,949 (12)	6138/96,866 (6)
Previous CABG (%)	3284/34,925 (9)	4234/108,366 (4)	2221/20,051 (11)	4646/97,015 (5)
STEMI (%)	10,841/38,406 (28)	50,370/119,165 (42)	5917/21,132 (28)	41,232/102,088 (40)
Good LV function (%)	9207/27,021 (34)	30,612/82,446 (37)	5088/15,474 (33)	26,993/73,452 (37)
Moderate LVSD (%)	6341/27,021 (23)	18,493/82,466 (22)	3586/15,474 (23)	16,020/73,452 (22)
Severe LVSD (%)	2615/27,021 (10)	6015/82,446 (7)	1478/15,474 (10)	4620/73,452 (6)
Cardiac arrest (%)	1914/37,244 (5)	7225/115,335 (6)	1152/20,657 (6)	6475/99,787 (6)

*Note*: Admission to cardiology ward is a composite of admission to coronary care unit (CCU) and general cardiology ward. Chronic renal failure is recorded in MINAP as those with serum creatinine chronically elevated above 200 micromol.

Abbreviations: BMI, body mass index; CABG, coronary artery bypass graft; CAD, coronary artery disease; CCF, congestive cardiac failure; COPD, chronic obstructive pulmonary disease; GRACE, global registry of acute coronary events; IQR, interquartile range; LVSD, left ventricular systolic dysfunction; MI, myocardial infarction.

**TABLE 2:** Management strategy and clinical outcome comparison for patients admitted with AMI according to socio‐economic status, stratified by presence of diabetes mellitus.

Variables	Quintile 1 (most deprived)		Quintile 5 (least deprived)	
	Diabetes mellitus (*n* = 38,406)	No DM (*n* = 119,165)	DM (*n* = 21,132)	No DM (*n* = 102,088)
Aspirin (%)	36,721/38,198 (96)	114,676/118,460 (97)	20,180/20,985 (96)	98,416/101,441 (97)
P2Y12 inhibitor (%)	31,768/37,096 (86)	97,757/114,553 (85)	18,008/20,583 (87)	87,176/99,249 (88)
Statins (%)	34,225/38,061 (90)	99,867/117,849 (84)	18,128/20,913 (87)	83,975/101,047 (83)
ACE inhibitors/ARB (%)	30,072/37,945 (79)	89,753/117,460 (76)	16,518/20,878 (79)	76,668/100,864 (76)
Beta blockers (%)	30,062/37,987 (79)	94,393/117,644 (80)	16,894/20,892 (81)	82,490/100,960 (82)
Coronary angiogram (%)	23,438/37,033 (63)	78,595/115,036 (68)	13,120/20,625 (64)	69,978/99,810 (70)
Percutaneous coronary intervention (%)	14,123/37,821 (37)	52,605/117,147 (45)	7605/20,808 (37)	45,604/100,311 (45)
CABG surgery (%)	1119/28,765 (4)	2353/89,529 (3)	604/16,352 (4)	1904/80,362 (2)
Revascularization (CABG surgery/PCI) (%)	15,198/37,821 (40)	54,860/117,147 (47)	8187/20,808 (39)	47,415/100,311 (47)
Inpatient mortality (%)	2497/38,406 (7)	6332/119,165 (5)	1593/21,132 (8)	5974/102,088 (65)
One‐year mortality (%)	7524/38,406 (20)	17,170/119,165 (14)	4529/21,132 (21)	15,019/102,088 (15)
Five‐year mortality (%) (Kaplan–Meier estimate)	44	30	44	30
Reinfarction (%)	485/33,682 (1)	1394/104,365 (1)	280/19,144 (1)	1302/92,262 (1)
Major bleeding (%)	631/36,929 (2)	1828/114,019 (2)	390/20,469 (2)	1654/98,592 (1)

*Note*: MACE is defined as composite endpoint of in‐hospital death and reinfarction. Chronic kidney disease is recorded in MINAP as those with serum creatinine chronically elevated above 200 micromol/L.

Abbreviations: ACE, angiotensin‐converting enzyme; ARB, angiotensin receptor blockers; CABG, coronary artery bypass graft; IV, intravenous; MRA, mineralocorticoid receptor antagonist; PCI, percutaneous coronary intervention; MACE, major adverse cardiovascular events.

Post‐estimation commands (*margins* in Stata) were used on an extracted dataset from our imputed dataset, following the running of a logistic regression model with 1‐year mortality as the outcome, adjusting for the variables mentioned earlier, with an interaction term included to assess the interaction of IMD quintile and DM status. Adjusted 1‐year mortality was plotted for the different combinations of IMD quintile and DM status. As a supplementary analysis, this adjusted 1‐year mortality analysis was repeated for three separate time periods, early (2005–2009), middle (2010–2014) and late (2015–2019), to illustrate whether our relationship between socio‐economic deprivation and DM status has changed over the study period. A supplementary analysis was undertaken, utilising one‐to‐one propensity score matching, for the same covariates as the Cox models in the main analysis, using the ‘psmatch2’ function on Stata, on a single dataset extracted from our 10 imputed datasets. This formed a population of 87,485 with DM and 87,485 without. We then repeated the same analysis on the matched dataset, clustering for matched pairs where appropriate. All analyses were conducted using Stata v18. Our graphical abstract was designed using a personal BioRender.com licence.

## 3 RESULTS

After applying study selection criteria on a total of 999,504 patients with AMI admitted to England and Wales hospitals from January 2005 up until March 2019, a total of 729,722 patients, 152,861 of whom had a known diagnosis of DM, were studied (21%) (Figure 1). Quintile 1 (Q1) refers to the most deprived quintile according to IMD score; Quintile 5 (Q5) refers to the least deprived throughout the results.

### 3.1 Baseline demographics of patients with AMI according to socio‐economic status and the presence of DM

Patients with DM from the most deprived quintile were younger (Q1: 69.8 years old vs. Q5: 75 years old), more likely to be of Asian ethnicity (Q1: 19% vs. Q5: 6%) and more likely to be current smokers than people with diabetes in the least deprived quintile (Q1: 28% vs. Q5: 13%) (Table 1). There was no difference in the proportion with severely impaired LV systolic function (Q1: 10% vs. Q5: 10%). The proportion of AMIs that were STEMIs was consistent across all quintiles for patients with DM at ~28%. When considering patients without DM from the most deprived regions, these were younger (Q1: 65.9 years old vs. Q5: 72.2 years old) and less likely to be of White ethnicity than the least deprived patients without DM (Q1: 91% vs. Q5: 97%) (Table 1). In patients without DM, the proportion of current smokers was highest in the most deprived quintile (Q1: 44% vs. Q5: 19%), as was the proportion of STEMI (Q1: 42% vs. Q5: 40%).

### 3.2 Baseline investigations and management of patients with AMI according to socio‐economic status and the presence of DM

Rates of receipt of aspirin (Q1: 96% vs. Q5: 96%), and ACE inhibitors/ARBs (Q1: 79% vs. Q5: 79%) were similar across quintiles for patients with DM (Table 2). Rates of prescription of statins (Q1: 90% vs. Q5: 87%) were higher in the most deprived quintile, whereas beta‐blocker prescription was higher in the more affluent quintile (Q1: 79% vs. Q5: 81%). Rates of invasive coronary angiography (ICA; Q1: 63% vs. Q5: 64%) and percutaneous coronary intervention (PCI; Q1: 37% vs. Q5: 37%) were similar across quintiles.

**FIGURE 2:**
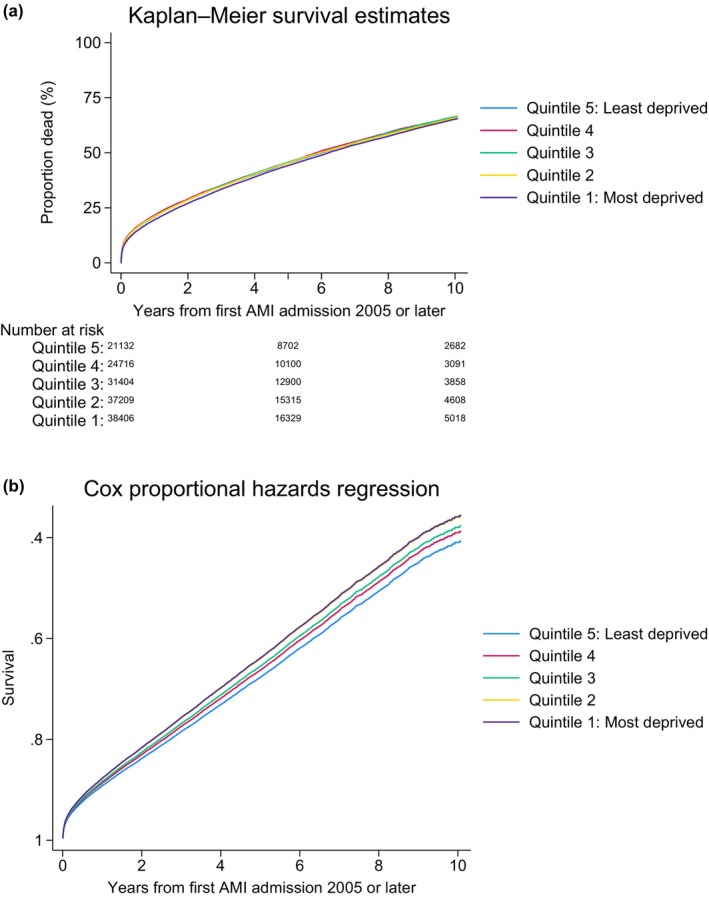
Unadjusted and adjusted survival curves for diabetes mellitus patients with AMI according to socio‐economic status. Adjusted survival calculated from Cox model adjusting for age, sex, year, hospital region, heart rate, blood pressure, comorbid conditions (hypertension, history of asthma or COPD, history of CVA or PVD, hypercholesterolaemia, family history of coronary artery disease, smoking history, previous AMI, previous PCI and previous CABG), cardiac arrest, LV systolic function, Killip classification and ischaemic ECG changes, medication strategy (warfarin, LMWH, UFH, glycoprotein B3A, aspirin, P2Y12 inhibitor, statin, beta‐blockers and ACE inhibitors), invasive angiography while an inpatient and revascularisation by PCI or CABG.

Kaplan–Meier estimates of 5‐year mortality were similar across IMD quintiles during the study period for patients with DM (Q1: 44% vs. Q5: 44%) (Figure 2). Rates of PCI for patients without DM (Q1: 45% vs. Q5: 45%) were similar across quintiles (Table 2). More deprived patients without DM were less likely to undergo ICA (Q1: 68% vs. Q5: 70%). Kaplan–Meier estimates of 5‐year mortality were unchanged between quintiles for patients without DM (Q1: 30% vs. Q5: 30%).

### 3.3 Survival analyses for patients with DM with AMI according to socio‐economic status

**FIGURE 3:**
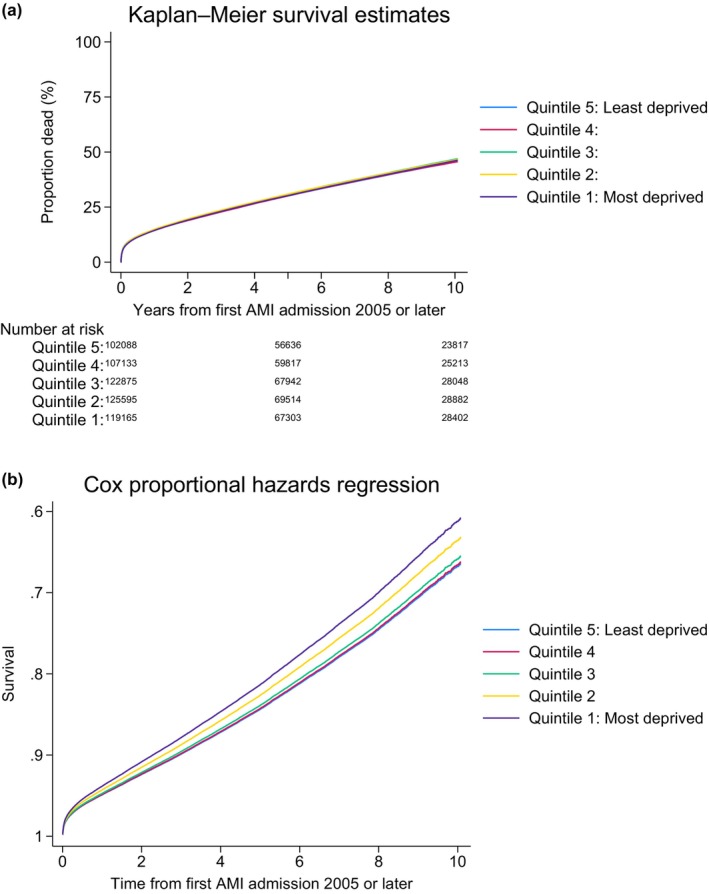
Unadjusted and adjusted survival curves for all AMI patients without diabetes mellitus according to socio‐economic status. Adjusted survival calculated from Cox‐model adjusting for age, sex, ethnicity (classified according to the MINAP categories; White, Black, Asian, Mixed and Other), year of admission, hospital region, admission heart rate, admission systolic blood pressure, comorbid conditions (hypertension, history of asthma or chronic obstructive pulmonary disease (COPD), history of cerebrovascular accident (CVA) or peripheral vascular disease (PVD), hypercholesterolaemia, family history of coronary artery disease, smoking history, previous AMI, history of angina, history of previous PCI and previous CABG), medication strategy (aspirin, P2Y12 inhibitor, LMWH, fondaparinux, warfarin, unfractionated heparin (UFH), glycoprotein B3A, ACE inhibitor/ARB, statin, beta‐blocker), cardiac arrest, left ventricular (LV) systolic function, Killip classification and ischaemic ECG changes and invasive coronary angiography, revascularisation by percutaneous coronary intervention (PCI) or coronary artery bypass grafts surgery (CABG).

In people with DM, 30‐day mortality risk was similar for those from most deprived compared to the most affluent backgrounds (adjusted hazard ratio (aHR): 1.01 (0.95–1.07), *p* = 0.846; Table 3). Risks of 1‐year (aHR: 1.05 (1.01–1.10), *p* = 0.010), 5‐year (aHR: 1.14 (1.11–1.17), *p* < 0.001) and overall mortality (aHR: 1.14 (1.12–1.17), *p* < 0.001) were higher for people in the most deprived quintile. Figure 3a,b show unadjusted and adjusted survival over our study period. Adjusted 1‐year mortality of patients with DM was highest in the second most deprived quintile, Q2: 19.1% (18.7%–19.4%) versus Q1: 18.8% (18.4%–19.1%) versus Q5: 17.8% (17.4%–18.2%), *p* < 0.001 (Table 5). The interaction between DM status and quintile of deprivation was significant at all quintiles (*p* < 0.001).

**TABLE 3:** Survival analysis for patients with diabetes mellitus with AMI according to socio‐economic status.

Outcome variables	Adjusted hazard ratio for patients compared to most affluent Quintile (Q5) according to IMD score (95% CIs)							
	Quintile 1 (most deprived)	*p*‐value	Quintile 2	*p*‐value	Quintile 3	*p*‐value	Quintile 4	*p*‐value
Primary outcomes								
Thirty‐day mortality	1.01 (0.95–1.07)	0.846	1.07 (1.01–1.14)	0.032	1.03 (0.96–1.09)	0.420	1.04 (0.97–1.11)	0.235
One‐year mortality	1.05 (1.01–1.10)	0.010	1.09 (1.05–1.13)	0.001	1.04 (1.00–1.08)	0.051	1.03 (0.98–1.07)	0.225
Five‐year mortality	1.14 (1.11–1.17)	<0.001	1.11 (1.09–1.14)	<0.001	1.07 (1.04–1.09)	<0.001	1.02 (1.00–1.05)	0.090
Overall mortality	1.14 (1.12–1.17)	<0.001	1.11 (1.09–1.14)	<0.001	1.07 (1.05–1.10)	<0.001	1.03 (1.00–1.07)	0.049

*Note*: Adjusted hazard ratios are presented with 95% CIs, adjusted for: age, sex, ethnicity (classified according to the MINAP categories; White, Black, Asian, Mixed and Other), year of admission, hospital region, admission heart rate, admission systolic blood pressure, co‐morbid conditions (hypertension, history of asthma or chronic obstructive pulmonary disease (COPD), history of cerebrovascular accident (CVA) or peripheral vascular disease (PVD), hypercholesterolaemia, family history of coronary artery disease, smoking history, previous AMI, history of angina, history of previous PCI and previous CABG), medication strategy (aspirin, P2Y12 inhibitor, LMWH, fondaparinux, warfarin, unfractionated heparin (UFH), glycoprotein B3A, ACE inhibitor/ARB, statin, beta blocker), cardiac arrest, left ventricular (LV) systolic function, Killip classification and ischaemic ECG changes, and invasive coronary angiography, revascularisation by percutaneous coronary intervention (PCI) or coronary artery bypass grafts surgery (CABG).

### 3.4 Survival analysis for patients without DM with AMI according to socio‐economic status

**FIGURE 4:**
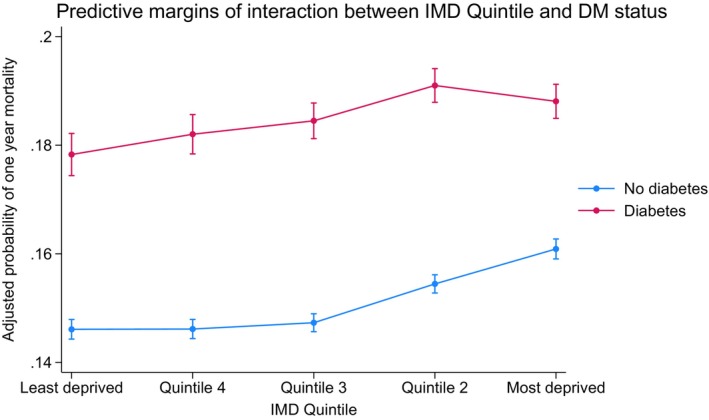
Margins plot showing interaction between diabetes status and IMD deprivation quintile on probability of 1‐year mortality. Adjusted 1‐year mortality calculated from logistic regression model, adjusting for age, sex, ethnicity (classified according to the MINAP categories; White, Black, Asian, Mixed and Other), year of admission, hospital region, admission heart rate, admission systolic blood pressure, comorbid conditions (hypertension, history of asthma or chronic obstructive pulmonary disease (COPD), history of cerebrovascular accident (CVA) or peripheral vascular disease (PVD), hypercholesterolaemia, family history of coronary artery disease, smoking history, previous AMI, history of angina, history of previous PCI and previous CABG), medication strategy (aspirin, P2Y12 inhibitor, LMWH, fondaparinux, warfarin, unfractionated heparin (UFH), glycoprotein B3A, ACE inhibitor/ARB, statin, beta‐blocker), cardiac arrest, left ventricular (LV) systolic function, Killip classification and ischaemic ECG changes, and invasive coronary angiography, revascularisation by percutaneous coronary intervention (PCI) or coronary artery bypass graft surgery (CABG).

Adjusted relative risk of mortality was highest in the most deprived quintile of patients without DM at 30 days (aHR: 1.05 (1.01–1.09), *p* = 0.009), 1 year (aHR: 1.12 (1.09–1.14), *p* < 0.001), 5 years (aHR: 1.18 (1.16–1.20), *p* < 0.001) and overall (aHR: 1.22 (1.20–1.23), *p* < 0.001) when compared to the least deprived quintile (Table 4). The absolute adjusted 1‐year mortality for patients without DM was highest in the most deprived quintile (Q1: 16.1% (15.9%–16.3%) vs. Q5: 14.6% (14.4%–14.8%) *p* < 0.001) (Table 5). Figure 4 shows the adjusted absolute 1‐year mortality risk according to socio‐economic and diabetes status—as socio‐economic deprivation status increases, so does mortality risk in people with and without DM. Figure S1 illustrates the temporal trends of the adjusted absolute 1‐year mortality, across three time periods. Adjusted 1‐year mortality has declined over the study period in all patients; the effect of socio‐economic deprivation on people without DM remains consistent, with a clear socio‐economic gradient; however, there is temporal variation in DM patients, with no variation according to socio‐economic status between 2010 and 2014, and a subtle relationship in 2005–2009 and 2015–2019.

**TABLE 4:** Survival analysis for patients without diabetes mellitus with AMI according to socio‐economic status.

Outcome variables	Adjusted hazard ratio for patients compared to most affluent Quintile (Q5) according to IMD score (95% CIs)							
	Quintile 1 (most deprived)	*p*‐value	Quintile 2	*p*‐value	Quintile 3	*p*‐value	Quintile 4	*p*‐value
Primary outcomes								
Thirty‐day mortality	1.05 (1.01–1.09)	0.009	1.03 (0.99–1.06)	0.098	0.99 (0.95–1.02)	0.430	0.98 (0.95–1.02)	0.320
One‐year mortality	1.12 (1.09–1.14)	<0.001	1.06 (1.04–1.09)	<0.001	1.00 (0.98–1.02)	0.968	0.99 (0.97–1.02)	0.582
Five‐year mortality	1.18 (1.16–1.20)	<0.001	1.11 (1.09–1.12)	<0.001	1.04 (1.02–1.05)	<0.001	1.01 (1.00–1.03)	0.104
Overall mortality	1.22 (1.20–1.23)	<0.001	1.13 (1.11–1.14)	<0.001	1.06 (1.04–1.07)	<0.001	1.02 (1.01–1.03)	0.004

*Note*: Adjusted hazard ratios are presented with 95% CIs, adjusted for: age, sex, ethnicity (classified according to the MINAP categories; White, Black, Asian, Mixed and Other), year of admission, hospital region, admission heart rate, admission systolic blood pressure, co‐morbid conditions (hypertension, history of asthma or chronic obstructive pulmonary disease (COPD), history of cerebrovascular accident (CVA) or peripheral vascular disease (PVD), hypercholesterolaemia, family history of coronary artery disease, smoking history, previous AMI, history of angina, history of previous PCI and previous CABG), medication strategy (aspirin, P2Y12 inhibitor, LMWH, fondaparinux, warfarin, unfractionated heparin (UFH), glycoprotein B3A, ACE inhibitor/ARB, statin, beta blocker), cardiac arrest, left ventricular (LV) systolic function, Killip classification and ischaemic ECG changes and invasive coronary angiography, revascularisation by percutaneous coronary intervention (PCI) or coronary artery bypass grafts surgery (CABG).

**TABLE 5:** Adjusted probability of one‐year mortality according to the interaction of 1‐year mortality with IMD Quintile and diabetes mellitus status.

Outcome variables	Adjusted probability of 1‐year mortality for patients according to interaction between socio‐economic status and diabetes mellitus status (95% CIs)	
Combinations of IMD quintile and DM status	Adjusted 1‐year mortality with 95% CIs (%)	*p*‐value for interaction
Quintile 1 (most deprived) and no DM	16.1 (15.9–16.3)	<0.001
Quintile 1 (most deprived) and DM	18.8 (18.4–19.1)	
Quintile 2 and no DM	15.4 (15.2–15.6)	<0.001
Quintile 2 and DM	19.1 (18.7–19.4)	
Quintile 3 and no DM	14.7 (14.6–14.9)	<0.001
Quintile 3 and DM	18.4 (18.1–18.8)	
Quintile 4 and no DM	14.5 (14.4–14.8)	<0.001
Quintile 4 and DM	18.2 (17.8–18.6)	
Quintile 5 (least deprived) and no DM	14.6 (14.4–14.8)	<0.001
Quintile 5 (least deprived) and DM	17.8 (17.4–18.2)	

*Note*: *p*‐values displayed are for interaction comparison within Quintile group according to presence of diabetes mellitus (DM). Adjusted 1‐year mortality presented with 95% CIs, adjusted for: age, sex, year, hospital region, heart rate, blood pressure, comorbid conditions (hypertension, history of asthma or COPD, history of CVA or PVD, hypercholesterolaemia, family history of coronary artery disease, smoking history, previous AMI, previous PCI and previous CABG), cardiac arrest, LV systolic function, Killip classification and ischaemic ECG changes, medication strategy (warfarin, LMWH, UFH, glycoprotein B3A, aspirin, P2Y12 inhibitor, statin, beta blockers and ACE inhibitors), invasive angiography while an inpatient and revascularisation by PCI or CABG.

### 3.5 Quality of care metrics with AMI according to socio‐economic status, stratified by the presence of DM

For NSTEMI patients with DM, the proportion of patients that underwent ICA within 72 hrs was slightly lower in people in the most deprived compared to the least deprived quintile (Q1: 55% vs. Q5: 57%) (Table S1). The mean opportunity‐based quality indicator (OBQI) score was higher in people with DM in the most deprived quintile compared to the least deprived quintile (Q1: 87.5 vs. Q5: 86.4) as was the rate of referral to cardiac rehabilitation at discharge (Q1: 81% vs. Q5: 77%).

For STEMI patients, patients with DM from the most deprived quintile had a slightly lower proportion of patients with a ‘Door‐to‐Balloon’ time under 120 min (Q1: 90% vs. Q5: 92%) (Table S2). Mean OBQI score was higher in the most deprived quintile of patients without DM (Q1: 91.3 vs. Q5: 90.3). Opportunity‐based quality indicator scores for both NSTEMI (Q1: 85.0 vs. Q5: 84.3) and STEMI patients without DM (Q1: 91.9 vs. Q5: 91.7) were consistent across quintiles (Tables S1 and S2).

## 4 DISCUSSION

Our nationwide cohort study assessing the importance of socio‐economic deprivation for outcomes following AMI patients with DM has a range of important implications. Firstly, we show that compared to those least deprived, patients with DM from regions of highest socio‐economic deprivation (according to IMD score) have a higher risk of mortality over a long‐term follow‐up (at 1 year, 5 years and overall (entire study period)), despite adjusting for a range of important covariates, including baseline demographics, common comorbidities and inpatient management strategy. However, the relative impact of deprivation on mortality risk post AMI is smaller in people with DM when compared to people without DM. Secondly, patients with DM from the most deprived quintile received excellent quality inpatient care, with high rates of GDMT prescription, comparable rates of ICA, PCI and higher rates of referral to cardiac rehabilitation services when compared to those with DM from the least deprived quintiles, defying the relationship that is typically seen between SES and inpatient AMI care outside of the United Kingdom (UK). Finally, in our assessment of the interaction of IMD quintile and DM status, although the adjusted 1‐year mortality of DM patients is higher throughout socio‐economic quintiles, the impact of socio‐economic status is less consistent in patients with DM, whereas there is a clearer relationship of increasing one‐year mortality in patients without DM as socio‐economic status declines.

Contemporary studies assessing the impact of socio‐economic status have important limitations in their applicability to the UK population. A large proportion of studies were undertaken in the United States (US), where the insurance‐based healthcare system introduces a clear cause of disparities in the quality of care and survival post‐AMI.^20^, ^21^ Interestingly, although disparities are mitigated by a universal healthcare system such as in the United Kingdom, this has been demonstrated to not be true, with socio‐economic disparities in access persisting within a national healthcare system.^22^, ^23^ Additionally, most studies have used in‐hospital clinical outcomes rather than longer‐term follow‐up as with our study. To our knowledge, this is the first major study on the impact of SES on the long‐term mortality of DM patients post AMI, and importantly contrasting that to the trends seen in a large cohort of patients without DM.

It is well established that DM has a significant impact on both short‐term and long‐term mortality post AMI. ^24^, ^25^ Previous studies from the United Kingdom have shown elevated risks for cardiovascular mortality in patients with DM compared to patients without DM, that increases with increasing deprivation.^26^ Similarly, US data have demonstrated elevated diabetes‐related and cardiovascular mortality in DM patients according to socio‐economic status.^10^, ^27^ It is well known that a range of important comorbidities that influence the risk of cardiovascular mortality are more likely to be present in the most socio‐economically deprived patients,^28^ but there is clearly an effect of socio‐economic status that extends beyond this, which is where an understanding of the social determinants of health is important. This comprises income disparity, educational status, neighbourhood factors and health accessibility, which combine to create adverse impacts on cardiovascular health.^4^, ^29^ Given that poorer socio‐economic status has been shown to influence AMI mortality previously^30^ here was a risk that patients with DM would be particularly high‐risk for socio‐economic disparity in AMI care and longer‐term outcomes. It was therefore surprising that the relationship between socio‐economic deprivation and long‐term mortality was stronger in patients without DM.

The reasons for this may, in part, lie in the characteristics of the most deprived cohort of patients without DM. This was a particularly young cohort, with a high proportion of important cardiovascular risk factors, such as being current smokers, having asthma and COPD and a higher proportion of ST‐elevation myocardial infarction. Patients with COPD are at higher risk of poorer outcomes post‐AMI, and frequent COPD exacerbations are associated with long‐term elevated cardiovascular risk,^31^ whereas rates of current smoking and levels of asthma and COPD were far lower in our patients with DM across the socio‐economic spectrum.

Quality of care in our study was excellent across the DM quintiles, with no significant differences according to SES for rates of prescription of GDMT, invasive investigations or revascularisation, which contrasts with US data that typically demonstrates poorer quality inpatient care according to socio‐economic status.^21^, ^32^ This could be reflective of the universal healthcare system in the United Kingdom, where insurance‐based disparities in access to high‐quality AMI care should be minimised, but may also be reflective of the significant age differences between the most deprived and affluent quintiles, as it is well demonstrated that younger AMI patients typically receive higher‐quality inpatient care.^33^ Additionally, the demographics of the DM cohort here may be important, with a significantly higher proportion of ethnic minority patients in the most deprived quintile. We have previously demonstrated that ethnic minority patients get higher‐quality inpatient care for NSTEMI^14^ in England and Wales. Access to healthcare is implicated as a key reason in the poorer outcomes of lower SES patients, with poorer rates of referral to secondary care from general practice for more deprived patients; for example,^34^ we suggest that a diagnosis of DM could be leading to more regular engagement with community and outpatient healthcare, alongside more aggressive risk factor management (reflected in the higher rates of statin prescription for patients with DM for example), which may mitigate socio‐economic disparities that are more pronounced in patients without DM. Additionally, there will have been changes in access to specialty cardiology care over the study period; we know that increasing proportions of UK patients received specialty cardiology care on a dedicated cardiology ward over 2005–2019,^35^ increased access to speciality care could be mitigating any discrepancy in outcomes in high‐risk people with DM.

Overall, the smaller DM‐related socio‐economic effect is a multifactorial observation, with the combination of relative youth, perception of DM as a significant AMI risk factor, high‐quality inpatient care and increased contact of patients with DM with outpatient and community medical services across the socio‐economic spectrum may be mitigating any impact of adverse SES on patients with DM.

Despite high‐quality inpatient care, mortality risk begins to increase at 1 year in patients with DM in the most deprived quintile, and one of the potential reasons for this is the suggested reduced long‐term compliance of patients with lower socio‐economic status with both cardiovascular medicines post‐acute coronary syndrome, likelihood of post‐acute heart failure admissions, and additionally with glucose‐lowering medications for DM.^36^, ^37^, ^38^ This could be a potential mechanism whereby early mortality is not increased, whereas long‐term mortality does increase, with non‐compliance with important medical therapy components such as ACE inhibitors, beta‐blockers and antiplatelet agents for example. Further research should focus on identifying the key disparities in both the care and follow‐up of patients from the most deprived regions of the United Kingdom. Particularly, attention needs to be directed towards the most socio‐economically deprived patients who do not have DM, and this is a significant cohort of patients for which care is not yet optimised.

## 5 STRENGTHS AND LIMITATIONS

There are several strengths to this study. Our analysis represents the largest study to date that looks at the impact of socio‐economic status on the outcomes of DM patients post‐AMI from a national healthcare service. The MINAP database encapsulates an almost complete record of AMI patients admitted in the United Kingdom and represents one of the largest national real‐world databases of this cohort of patients in the world, including those that are high risk and have multiple comorbid illnesses, such that they are either not included or under‐represented in clinical trials.

Despite these strengths, there are several important limitations common to observational studies of this type. The MINAP data collection shares the weakness of other national registries, including self‐reporting of adverse events where there is no external validation of these. Although the MINAP dataset included important clinical and demographic variables of interest, there are limitations to the data collected. For instance, the database does not capture frailty score or index, severity of coronary artery disease, procedural details such as whether PCI was single or multi‐vessel, contraindications to medications or an exhaustive list of comorbid conditions. Our linkage of the registry to IMD score gives a comprehensive picture of deprivation for each record in our registry, but notably our data linkage does not show the individual metrics that make up the IMD score; therefore, we are unable to comment on individual metrics such as educational status or household income, for example. Additionally, we acknowledge that across the DM cohort, there are higher proportions of modifiable cardiovascular risk factors such as hypercholesterolemia and higher BMI across the socio‐economic spectrum, and our suggestion that a diagnosis of DM is mitigating the socio‐economic effect on AMI mortality could be more complex and reflect the entire metabolic syndrome rather than DM alone. Ethnicity is an important consideration in studies of people with DM; we acknowledge that there is a high proportion of missing data in the ethnicity variable in the MINAP registry. Furthermore, where ethnicity data are available, it is in crude categories such as White, Black and Asian, where these can be heterogeneous groups with a range of healthcare outcomes.

Finally, although our Cox and post‐estimate models correct for a comprehensive range of covariates, covering demographic, comorbidity and management strategy, residual confounding is possible. Additionally, our registry does not record type of DM; therefore, we are unable to discern between type 1 and type 2 diabetes mellitus (T1DM and T2DM). Additionally, we lack data on the detail of the medical management of diabetes, not knowing whether patients are on the newer generation anti‐hyperglycaemic agents such as SGLT2 inhibitors or GLP‐1 receptor agonists.

## 6 CONCLUSION

Patients from England and Wales with DM have poorer long‐term survival at 1 year, 5 years and over our entire study period post‐AMI when from the most socio‐economically deprived regions compared to the most affluent; however, there is no significant difference in the risk of 30‐day mortality. Patients without DM from the most deprived regions are at elevated risk of 30‐day, 1‐year, 5‐year and overall mortality, and there is more of an effect on mortality between the most deprived and affluent than seen in patients with DM. Further work is required to understand the disparities in long‐term survival according to socio‐economic status, with particular focus on patients without DM from the most socio‐economically deprived regions.

## FUNDING INFORMATION

This research is funded by the National Institute for Health and Care Research (NIHR), NIHR Birmingham Biomedical Research Centre, Birmingham, UK and supported by the NIHR Manchester Biomedical Research Centre. The views expressed are those of the author(s) and not necessarily those of the NIHR or the Department of Health and Social Care. MR is funded by an NIHR DSE award and AMS grant (Academy of Medical Sciences) (SGL025/1064). SM is funded by a Wellcome Trust Career Development Award (223024/Z/21/Z) and is supported by the NIHR Imperial Biomedical Research Centre, Sciences (SGL025/1064). KK is supported by the National Institute for Health Research (NIHR) Applied Research Collaboration East Midlands (ARC EM), NIHR Global Research Centre for Multiple Long Term Conditions, Multiple Long‐Term Conditions Cross‐NIHR Collaboration, NIHR Leicester Biomedical Research Centre (BRC) and the British Heart Foundation (BHF) Centre of Excellence.

## CONFLICT OF INTEREST STATEMENT

NW's research fellowship is funded by Abbott Vascular.

## ETHICS STATEMENT

Secondary use of anonymised MINAP dataset for research purposes is authorised under NHS research governance arrangements and further supported under section 251 of NHS act 2006 (NIGB: ECC1‐06(d)/ 2011), which allows researchers to use patient information collected within the dataset for medical research without patient consent. Therefore, a formal ethical approval was not sought for this study. The study underwent formal ethical approval for the data linkages of MINAP and ONS registries. The ethical approval was provided by the Health Research Authority and the Health and Care Research Wales and the Confidentiality Advisory Group, which is an independent body that provides expert advice on the use of confidential patient information.

## Supporting information


Data S1.

## Data Availability

The authors do not have authorisation to share the data, but it can be accessed through contacting the National Institute for Cardiovascular Outcomes Research (NICOR) upon approval.
