# Supplementary material for: The impact of socio‐economic deprivation on the long‐term survival of people with diabetes and acute myocardial infarction: A nationwide cohort study
Source: Diabet Med. 2025 Jul 28;42(11):e70111. doi: 10.1111/dme.70111 (PMC12535319; doi:10.1111/dme.70111)
Supplement: Supplementary file 1 — Data S1. [file DME-42-e70111-s001.docx]

**Supplementary Table 1: Demographic comparison for patients admitted with AMI according to socioeconomic status, stratified by presence of diabetes mellitus**

| **Variables** | | **Quintile 1 (most deprived)** | | **Quintile 2** | | **Quintile 3** | | **Quintile 4** | | **Quintile 5 (most affluent)** | |
| --- | --- | --- | --- | --- | --- | --- | --- | --- | --- | --- | --- |
|  | **Diabetes Mellitus (n= 38,406)** | | **No DM (n= 119,165)** | **DM (n= 37,209)** | **No DM (n= 125,595)** | **DM (n=31,404)** | **No DM (n= 122,875)** | **DM (n= 24,716)** | **No DM (n= 107,133)** | **DM (n=21,132)** | **No DM (n= 102,088)** |
| **Age, years, median (IQR)** | 69.8 (59.7-78.2) | | 65.9 (54.7-77.7) | 71.7 (61.7-79.7) | 68.7 (57.2-77.7) | 73.3 (63.9-80.8) | 70.5 (59.5-81) | 74.4 (65.3-81.6) | 71.2 (60.3-81.3) | 75.2 (66.3-82.2) | 72.2 (61.3-81.9) |
| **Female (%)** | 14,386/38,406 (37) | | 41,201/119,165 (35) | 13,306/37,209 (36) | 42,902/125,595 (34) | 10,606/31,404 (34) | 41,279/122,875 (34) | 8,242/24,716 (33) | 35,278/107,133 (33) | 6,824/21,132 (32) | 33,274/102,088 (33) |
| **BMI, median [IQR]** | 29.2 (25.6-33.5) | | 26.9 (23.7-30.5) | 28.7 (25.2-32.9) | 26.7 (23.7-30.1) | 28.5 (25.2-32.5) | 26.6 (23.7-29.8) | 28.3 (25.1-32.1) | 26.5 (23.7-29.6) | 28.0 (24.8-31.7) | 26.3 (23.6-29.4) |
| **Ethnicity- White (%)** | 16,456/21,193 (78) | | 54,273/59,510 (91) | 16,171/20,004 (81) | 58,068/62,594 (93) | 15,010/17,074 (88) | 58,657/61,505 (95) | 12,202/13,325 (91) | 51,836/52,527 (97) | 10,555/11,368 (93) | 49,795/51,137 (97) |
| **Ethnicity –Asian (%)** | 4,092/21,193 (19) | | 4,263/59,510 (7) | 3,219/20,004 (16) | 3,579/62,594 (6) | 1,764/17,074 (11) | 2,330/61,505 (4) | 1,003/13,325 (8) | 1,393/53,527 (3) | 737/11,368 (6) | 1,116/51,137 (2) |
| **Ethnicity –Black (%)** | 578/21,293 (3) | | 806/59,510 (1) | 525/20,004 (3) | 782/62,594 (1) | 248/17,074 (1) | 399/61,505 (1) | 91/13,325 (1) | 209/53,527 (0) | 54/11,368 (0) | 153/51,137 (0) |
| **Ethnicity –Mixed (%)** | 67/21,293 (0) | | 168/59,510 (0) | 99/20,004 (0) | 119/62,594 (0) | 52/17,074 (0) | 119/61,505 (0) | 29/13,325 (0) | 89/53,527 (0) | 22/11,368 (0) | 73/51,137 (0) |
| **Basal crepitations (%)** | 3,157/19,021 (17) | | 6,317/52,950 (12) | 3,271/18,921 (17) | 7,147/58,122 (13) | 2,675/15,870 (17) | 6,753/56,058 (12) | 2,157/12,460 (17) | 5,561/48,987 (11) | 1,731/10,610 (17) | 5,149/47,145 (11) |
| **Pulmonary oedema (%)** | 1,582/19,021 (8) | | 2,246/52,950 (4) | 1,560/18,921 (8) | 2,293/58,122 (4) | 1,270/15,870 (8) | 2,223/56,058 (4) | 1,001/12,460 (8) | 2,035/48,987 (4) | 840/10,610 (8) | 1,913/47,145 (4) |
| **Cardiogenic shock (%)** | 339/19,021 (2) | | 810/52,950 (2) | 364/18,921 (2) | 937/58,122 (2) | 276/15,870 (2) | 853/56,058 (2) | 173/12,460 (1) | 746/48,987 (2) | 164/10,610 (2) | 680/47,145 (1) |
| **High risk GRACE score >140 (%)** | 12,135/18,327 (66) | | 28,785/50,877 (57) | 12,581/18,135 (69) | 33,711/55,351 (61) | 10,905/15,137 (72) | 34,046/53,289 (64) | 8,683/11,905 (73) | 30,261/46,589 (65) | 7,585/10,145 (75) | 29,807/44,781 (67) |
| **Intermediate risk GRACE score 109-140 (%)** | 4,583/18,327 (25) | | 15,036/50,877 (30) | 4,162/18,135 (23) | 15,352/55,351 (28) | 3,230/15,137 (21) | 13,995/53,28926) | 2,543/11,905 (21) | 12,013/46,589 (26) | 2,009/10,145 (20) | 11,159/44,781 (25) |
| **Low risk GRACE score <109 (%)** | 1,609/18,327 (9) | | 7,056/50,877 (14) | 1,392/18,135 (8) | 6,288/55,351 (11) | 1,002/15,137 (7) | 5,248/53,289 (10) | 679/11,905 (6) | 4,315/46,589 (9) | 551/10,145 (5) | 3,815/44,781 (9) |
| **ECG ST changes (%)** | 31,267/36,996 (85) | | 100,300/115,222 (87) | 30,422/36,032 (84) | 105,360/121,829 (86) | 25,547/30,362 (84) | 103,113/119,276 (86) | 20,188/23,847 (85) | 90,046/103,649 (87) | 17,253/20,319 (85) | 85,469/98,371 (87) |
| **Previous smoker (%)** | 12,554/36,398 (34) | | 31,777/50,580 (28) | 12,918/34,826 (37) | 37,484/119,680 (31) | 11,715/29,371 (40) | 39,064/116,603 (34) | 9,510/23,061 (41) | 35,055/101,847 (34) | 7,993/19,631 (41) | 33,574/96,476 (35) |
| **Current smoker (%)** | 10,178/36,398 (28) | | 50,580/114,461 (44) | 7,592/34,826 (22) | 41,064/119,680 (34) | 5,119/29,371 (17) | 31,751/116,603 (27) | 3,535/23,061 (15) | 23,574/101,847 (23) | 2,523/19,631 (13) | 17,974/96,476 (19) |
| **CCF (%)** | 3,219/34,817 (9) | | 4,854/108,109 (4) | 3,225/34,556 (9) | 5,155/116,387 (4) | 2,687/29,264 (9) | 25,110/114,046 (4) | 2,044/22,980 (9) | 4,274/99,781 (4) | 1,722/19,860 (9) | 4,029/96,309 (4) |
| **Hypercholesterolemia (%)** | 16,736/34,829 (48) | | 30,460/107,750 (28) | 16,253/34,556 (47) | 32,739/115,759 (28) | 13,179/29,224 (45) | 31,296/113,910 (27) | 10,225/22,889 (45) | 27,842/99,484 (28) | 8,818/19,788 (45) | 27,294/95,713 (29) |
| **Cerebrovascular disease (%)** | 4,367/34,837 (13) | | 8,242/108,318 (8) | 4,210/34,657 (12) | 8,617/116,551 (7) | 3,460/29,286 (12) | 8,377/114,261 (7) | 2,579/23,013 (11) | 7,074/99,9857) | 2,345/19,913 (12) | 6,733/96,342 (7) |
| **History of angina (%)** | 12,687/35,081 (36) | | 22,565/108,744 (21) | 11,785/34,784 (34) | 23,520/117,247 (20) | 9,717/29,502 (33) | 22,578/115,406 (20) | 7,634/23,156 (33) | 19,242/100,719 (19) | 6,308/19,990 (32) | 18,109/96,875 (19) |
| **Peripheral vascular disease (%)** | 2,874/34,567 (8) | | 4,245/107,462 (4) | 2,643/34,348 (8) | 3,993/115,538 (3) | 2,241/29,066 (8) | 3,753/113,352 (3) | 1,775/22,803 (7) | 3,101/98,975 (3) | 1,514/19,715 (8) | 2,821/95,306 (3) |
| **Chronic renal failure (%)** | 4,185/34,786 (12) | | 4,319/108,016 (4) | 4,180/34,495 (12) | 4,917/116,238 (4) | 3,488/29,201 (12) | 4,903/113,879 (4) | 2,798/22,938 (12) | 4,302/99,723 (4) | 2,393/19,855 (12) | 4,104/96,259 (4) |
| **Hypertension (%)** | 24,151/35,563 (68) | | 47,078/109,803 (43) | 24,341/35,301 (69) | 53,062/118,240 (45) | 20,376/29,898 (68) | 52,813/116,442 (45) | 15,840/23,436 (68) | 46,341/101,507 (46) | 13,775/20,248 (69) | 45,205/97,647 (46) |
| **Asthma / COPD (%)** | 7,477/34,782 (22) | | 20,502/107,867 (19) | 6,278/34,515 (18) | 18,503/115,977 (16) | 4,589/29,170 (16) | 16,017/113,636 (14) | 3,432/22,899 (15) | 12,901/99,235 (13) | 2,604/19,785 (13) | 11,241/95,492 (12) |
| **Family history of CAD (%)** | 8,275/27,954 (30) | | 30,686/90,237 (34) | 7,432/27,718 (27) | 30,748/96,941 (32) | 6,236/23,371 (27) | 28,926/94,759 (31) | 4,938/18,466 (27) | 26,258/83,832 (31) | 4,288/15,958 (27) | 25,014/80,583 (30) |
| **Previous AMI (%)** | 10,365/35,289 (29) | | 17,642/109,503 (16) | 9,782/35,139 (28) | 18,270/118,612 (15) | 7,954/29,789 (27) | 17,277/116,747 (15) | 6,260/23,356 (27) | 14,818/101,561 (15) | 5,134/20,144 (25) | 13,758/97,510 (14) |
| **Previous PCI (%)** | 4,206/34,814 (12) | | 6,336/108,233 (6) | 4,275/34,539 (12) | 7,093/116,560 (6) | 3,618/29,289 (12) | 7,130/114,682 (6) | 2,780/23,023 (12) | 6,365/100,158 (6) | 2,329/19,949 (12) | 6,138/96,866 (6) |
| **Previous CABG (%)** | 3,284/34,925 (9) | | 4,234/108,366 (4) | 3,541/34,711 (10) | 4,992/116,672 (4) | 3,149/29,402 (11) | 5,239/114,832 (5) | 2,438/23,106 (11) | 4,785/100,308 (5) | 2,221/20,051 (11) | 4,646/97,015 (5) |
| **STEMI (%)** | 10,841/38,406 (28) | | 50,370/119,165 (42) | 10,257/37,209 (28) | 51,082/125,595 (41) | 8,660/31,404 (28) | 48,790/122,875 (40) | 6,827/24,716 (28) | 43,395/107,133 (41) | 5,917/21,132 (28) | 41,232/102,088 (40) |
| **Heart rate, bpm, median (IQR)** | 83 (70-98) | | 78 (66-92) | 82 (70-97) | 78 (66-91) | 81 (70-96) | 77 (65-90) | 81 (69-96) | 76 (65-90) | 80 (68-95) | 76 (65-90) |
| **Systolic blood pressure, median (IQR)** | 138 (120-157) | | 136 (118-155) | 138 (120-158) | 136 (119-155) | 139 (120-158) | 137 (120-156) | 139 (120-158) | 138 (120-157) | 139 (120-158) | 138 (120-157) |
| **Good LV function (%)** | 9,207/27,021 (34) | | 30,612/82,446 (37) | 9,305/26,881 (35) | 33,421/88,906 (38) | 7,598/22,622 (34) | 31,861/86,383 (37) | 6,056/18,042 (34) | 28,619/76,939 (37) | 5,088/15,474 (33) | 26,993/73,452 (37) |
| **Moderate LVSD (%)** | 6,341/27,021 (23) | | 18,493/82,466 (22) | 6,443/26,881 (24) | 19,626/88,906 (22) | 5,381/22,622 (24) | 18,853/86,383 (22) | 4,302/18,042 (24) | 16,919/76,939 (22) | 3,586/15,474 (23) | 16,020/73,452 (22) |
| **Severe LVSD (%)** | 2,615/27,021 (10) | | 6,015/82,446 (7) | 2,665/26,881 (10) | 6,058/88,906 (7) | 2,232/22,622 (10) | 5,768/86,383 (7) | 1,730/18,042 (10) | 4,909/76,939 (6) | 1,478/15,474 10) | 4,620/73,452 (6) |
| **Cardiac arrest (%)** | 1,914/37,244 (5) | | 7,225/115,335 (6) | 11,940/36,385 (5) | 7,897/122,792 (6) | 1,654/30,772 (5) | 7,477/120,291 (6) | 1,310/24,225 (5) | 6,509/104,859 (6) | 1,152/20,657 (6) | 6,475/99,787 (6) |

CABG; coronary artery bypass graft, LVSD; left ventricular systolic dysfunction, CAD; coronary artery disease, COPD; chronic obstructive pulmonary disease, MI; myocardial infarction, CCF; congestive cardiac failure, BMI; body mass index, GRACE; global registry of acute coronary events, IQR; interquartile range. Admission to cardiology ward is a composite of admission to coronary care unit (CCU) and general cardiology ward. Chronic renal failure is recorded in MINAP as those with serum creatinine chronically elevated above 200 micromo

**Supplementary Table 2 Management strategy and clinical outcome comparison for patients admitted with AMI according to socioeconomic status, stratified by presence of diabetes mellitus**

| **Variables** | | **Quintile 1 (most deprived)** | | | **Quintile 2** | | | **Quintile 3** | | **Quintile 4** | | | **Quintile 5 (most affluent)** | | |
| --- | --- | --- | --- | --- | --- | --- | --- | --- | --- | --- | --- | --- | --- | --- | --- |
|  | **Diabetes Mellitus (n= 38,406)** | | **No DM (n= 119,165)** | **DM (n= 37,209)** | | **No DM (n= 125,595)** | **DM (n=31,404)** | | **No DM (n= 122,875)** | | **DM (n= 24,716)** | **No DM (n= 107,133)** | | **DM (n=21,132)** | **No DM (n= 102,088)** |
| **Low molecular weight heparin (%)** | 16,338/30,015 (55) | | 49,359/95,048 (52) | 17,540/31,751 (55) | | 57,423/106,593 (54) | 15,080/26,744 (56) | | 57,210/104,218 (55) | | 12,181/21,283 (57) | 50,943/92,363 (55) | | 10,827/18,527 (58) | 50,853/89,800 (57) |
| **Fondaparinux (%)** | 9,269/25,955 (36) | | 24,216/79,488 (30) | 9,442/27,803 (34) | | 27,190/90,544 (30) | 8,032/23,429 (34) | | 27,107/88,615 (31) | | 6,368/18,673 (34) | 23,773/78,922 (30) | | 5,448/16,356 (33) | 23,098/77,295 (30) |
| **Warfarin (%)** | 1,866/29,680 (6) | | 4,334/93,755 (5) | 2,020/31,375 (6) | | 5,033/104,896 (5) | 1,831/26,305 (7) | | 5,198/101,982 (5) | | 1,458/20,984 (7) | 4,585/90,831 (5) | | 1,304/18,322 (7) | 4,687/88,614 (5) |
| **Glycoprotein 2b/3a inhibitor (%)** | 2,037/30,125 (6) | | 8,939/95,125 (9) | 2,050/31,847 (6) | | 10,166/107,007 (10) | 1,775/26,927 (6) | | 9,423/104,812 (9) | | 1,352/21,377 (6) | 8,307/92,539 (9) | | 1,286/18,598 (7) | 8,517/90,010 (9) |
| **IV Nitrate (%)** | 5,208/29,627 (18) | | 14,832/93,706 (16) | 5,697/31,404 (18) | | 18,099/104,942 (17) | 4,779/26,314 (18) | | 17,943/102,025 (18) | | 3,684/20,985 (18) | 16,215/90,869 (18) | | 3,378/18,362 (18) | 15,865/88,687 (18) |
| **MRA (%)** | 2,178/22,059 (10) | | 4,653/66,064 (7) | 2,262/23,912 (9) | | 5,310/75,708 (7) | 1,889/20,084 (9) | | 5,283/74,272 (7) | | 1,618/16,124 (10) | 4,737/66,320 (7) | | 1,345/14,036 (10) | 4,692/65,325 (7) |
| **Aspirin (%)** | 36,721/38,198 (96) | | 114,676/118,460 (97) | 35,548/36,983 (96) | | 120,899/124,969 (97) | 30,025/31,212 (96) | | 118,214/122,152 (97) | | 23,591/24,545 (96) | 103,326/106,522 (97) | | 20,180/20,985 (96) | 98,416/101,441 (97) |
| **P2Y12 inhibitor (%)** | 31,768/37,096 (86) | | 97,757/114,553 (85) | 31,220/36,250 (86) | | 105,310/122,083 (86) | 26,505/30,529 (87) | | 103,282/118,755 (87) | | 20,887/24,026 (87) | 90,874/103,788 (88) | | 18,008/20,583 (87) | 87,176/99,249 (88) |
| **Statins (%)** | 34,225/38,061 (90) | | 99,867/117,849 (84) | 32,587/36,899 (88) | | 103,915/124,410 (84) | 27,219/31,107 (88) | | 101,166/121,614 (83) | | 21,415/24,476 (87) | 88,648/106,093 (84) | | 18,128/20,913 (87) | 83,975/101,047 (83) |
| **ACE inhibitors/ARB (%)** | 30,072/37,945 (79) | | 89,753/117,460 (76) | 28,996/36,839 (79) | | 93,735/124,159 (76) | 24,451/31,064 (79) | | 91,343/121,341 (75) | | 19,208/24,435 (79) | 80,509/105,866 (76) | | 16,518/20,878 (79) | 76,668/100,864 (76) |
| **Beta-Blockers (%)** | 30,062/37,987 (79) | | 94,393/117,644 (80) | 29,463/36,838 (80) | | 99,931/124,257 (80) | 24,833/31,078 (80) | | 97,570/121,495 (80) | | 19,592/24,446 (80) | 86,330/105,974 (81) | | 16,894/20,892 (81) | 82,490/100,960 (82) |
| **Coronary angiogram (%)** | 23,438/37,033 (63) | | 78,595/115,036 (68) | 22,857/35,988 (64) | | 83,615/121,859 (69) | 19,450/30,497 (64) | | 82,329/119,660 (69) | | 15,164/24,058 (63) | 72,641/104,403 (70) | | 13,120/20,625 (64) | 69,978/99,810 (70) |
| **Percutaneous coronary intervention (%)** | 14,123/37,821 (37) | | 52,605/117,147 (45) | 13,574/36,555 (37) | | 55,706/123,145 (45) | 11,186/30,806 (36) | | 53,672/120,594 (45) | | 8,725/24,288 (36) | 47,511/105,135 (45) | | 7,605/20,808 (37) | 45,604/100,311 (45) |
| **CABG surgery (%)** | 1,119/28,765 (4) | | 2,353/89,529 (3) | 1,179/28,478 (4) | | 2,487/96,694 (3) | 952/24,056 (4) | | 2,320/95,015 (2) | | 718/19,020 (4) | 2,097/83,341 (3) | | 604/16,352 (4) | 1,904/80,362 (2) |
| **Revascularization (CABG surgery/PCI) (%)** | 15,198/37,821 (40) | | 54,860/117,147 (47) | 14,719/36,555 (40) | | 58,078/123,145 (47) | 12,111/30,806 (39) | | 55,889/120,594 (46) | | 9,411/24,288 (39) | 49,518/105,135 (47) | | 8,187/20,808 (39) | 47,415/100,311 (47) |
| **Inpatient mortality (%)** | 2,497/38,406 (7) | | 6,332/119,165 (5) | 2,696/37,209 (7) | | 7,120/125,595 (6) | 2,237/31,404 (7) | | 6,866/122,875 (6) | | 1,836/24,716 (7) | 5,829/107,133 (5) | | 1,593/21,132 (8) | 5,974/102,08865) |
| **One year mortality (%)** | 7,524/38,406 (20) | | 17,170/119,165 (14) | 7,891/37,209 (21) | | 18,718/125,595 (15) | 6,626/31,404 (21) | | 18,003/122,875 (15) | | 5,348/24,716 (22) | 15,406/107,133 (14) | | 4,529/21,132 (21) | 15,019/102,088 (15) |
| **Five-year mortality (%) (Kaplan-Meier Estimate)** | **44** | | **30** | **45** | | **31** | **45** | | **31** | | **45** | **30** | | **44** | **30** |
| **Reinfarction (%)** | 485/33,682 (1) | | 1,394/104,365 (1) | 489/33,278 (1) | | 1,550/112,274 (1) | 414/28,248 (1) | | 1,626/110,221 (1) | | 355/22,196 (2) | 1,392/96,221 (1) | | 280/19,144 (1) | 1,302/92,262 (1) |
| **Major bleeding (%)** | 631/36,929 (2)_ | | 1,828/114,019 (2) | 716/35,909 (2) | | 2,073/120,890 (2) | 563/30,253 (2) | | 1,903/118,035 (2) | | 457/23,787 (2) | 1,779/103,027 (2) | | 390/20,469 (2) | 1,654/98,592 (1) |

IV**;** intravenous**,** MRA**;** mineralocorticoid receptor antagonist**,** ACE**:** angiotensin-converting-enzyme**,** ARB**;** angiotensin receptor blockers**,** CABG**;** coronary artery bypass graft**,** PCI**;** percutaneous coronary intervention and MACE**;** major adverse cardiovascular events. MACE is defined as composite endpoint of in-hospital death and reinfarction. **Chronic kidney disease** is recorded in MINAP as those with serum creatinine chronically elevated above 200 micromol/L.

**Supplementary Table 3: Quality Indicators for NSTEMI patients according to socioeconomic status, stratified by presence of diabetes mellitus (DM) (ESC ACVC and OBQI)**

| **Variables** | **Quintile 1 (most deprived)** | | **Quintile 2)** | | **Quintile 3** | | **Quintile 4** | | **Quintile 5 (most affluent)** | |
| --- | --- | --- | --- | --- | --- | --- | --- | --- | --- | --- |
|  | **Diabetes Mellitus (n= 27,565** | **No DM (n= 68,795)** | **DM (n= 26,952** | **No DM (n= 74,513)** | **DM (n= 22,744)** | **No DM (n= 73,905)** | **DM (n= 17,889)** | **No DM (n= 63,738)** | **DM (n= 15,215)** | **No DM (n= 60,856)** |
| **Coronary Angiography received within 72 hours (%)** | 5,202/10,115 (51) | 14,897/25,594 (58) | 4,919/9,874 (50) | 16,084/27,720 (58) | 4,260/8,459 (50) | 16,657/28,544 (58) | 3,365/6,715 (50) | 15,002/25,374 (59) | 3,135/5,794 (54) | 15,105/24,844 (61) |
| **LV Function recorded in notes (%)** | 12,391/19,249 (64) | 28,733/46,837 (61) | 12,739/19,271 (66) | 20,414/51,954 (62) | 10,610/16,323 (65) | 31,127/51,188 (61) | 8,367/12,994 (64) | 27,763/45,242 (61) | 6,923/11,063 (63) | 26,379/43,483 (61) |
| **Fondaparinux or LMWH received (%)** | 18,708/21,377 (88) | 48,489/54,294 (89) | 19,699/22,901 (86) | 55,712/63,114 (88) | 16,646/19,293 (86) | 55,243/62,340 (89) | 13,229/15,284 (87) | 48,423/54,645 (89) | 11,437/13,202 (87) | 47,129/53,206 (89) |
| **DAPT received on discharge (%)** | 21,910/26,676 (82) | 54,004/66,210 (82) | 21,813/26,304 (83) | 60,093/72,570 (83) | 18,512/22,140 (84) | 59,693/71,459 (84) | 14,519/17,375 (84) | 52,105/61,710 (84) | 12,466/14,796 (84) | 50,041/59,056 (85) |
| **ACEi or ARB on discharge for those with moderate and severe LVSD (%)** | 4,456/5,520 (81) | 7,792/10,486 (74) | 4,617/5,760 (80) | 8,712/11,670 (75) | 3,862/4,882 (79) | 8,408/11,279 (75) | 3,052/3,848 (79) | 7,543/10,041 (75) | 2,536/3,183 (80) | 7,071/9,561 (74) |
| **Beta Blocker on discharge for those for those with moderate and severe LVSD (%)** | 4,512/5,529 (82) | 8,392/10,521 (80) | 4,844/5,766 (84) | 9,561/11,686 (82) | 4,023/4,884 (82) | 9,294/11,300 (82) | 3,205/3,849 (83) | 8,335/10,055 (83) | 2,657/3,186 (83) | 7,933/9,582 (83) |
| **Composite All/None score* (%)** | 19,945/26,672 (75) | 45,475/66,250 (69) | 19,550/26,299 (74) | 49,821/72,587 (69) | 16,333/22,123 (74) | 49,336/71,583 (69) | 12,835/17,368 (74) | 43,158/61,782 (70) | 10,880/14,786 (74) | 41,112/59,077 (70) |
| **Composite All/None score for those with moderate and severe LVSD (%)** | 4,301/5,503 (78) | 7,486/10,479 (71) | 4,518/5,732 (79) | 8,338/11,636 (72) | 3,727/4,868 (77) | 8,205/11,251 (73) | 2,975/3,836 (78) | 7,283/10,030 (73) | 2,425/3,171 (76) | 6,794/9,546 (71) |
| **Mean OBQI score** | 85.5 | 83.0 | 84.5 | 81.9 | 84.4 | 82.1 | 84.5 | 82.8 | 84.7 | 82.7 |
| **Cardiac rehabilitation (%)** | 19,482/24,716 (79) | 49,805/61,769 (81) | 18,063/24,161 (75) | 52,070/67,261 (77) | 15,378/20,348 (76) | 51,996/66,827 (78) | 12,252/16,141 (76) | 45,838/57,920 (79) | 10,507/13,819 (76) | 44,088/55,709 (79) |

ESC; European Society of Cardiology, ACVC; Association for Acute Cardiovascular Care, GRACE; global registry of acute coronary events, CRUSADE; can rapid risk stratification of unstable angina patients suppress adverse outcomes with early implementation of the ACC/AHA guidelines, LV; left ventricle, EF; ejection fraction, LMWH; low molecular weight heparin, DAPT; dual antiplatelet therapy, ACEi/ARB; angiotensin converting enzyme inhibitor/angiotensin receptor blockers, LVSD; left ventricular systolic dysfunction, N/A; Not Available.

*Composite score of receipt of low dose aspirin, P2Y_12_ inhibition and statin.

**Opportunity based QI (The score consisted of 6 evidence-based processes of care: the prescription of aspirin, thienopyridine inhibitor, β-blocker, angiotensin converting enzyme inhibitor (ACEi), HMG CoA reductase enzyme inhibitor (statin) and enrolment onto a cardiac rehabilitation programme at the time of discharge). The OBCS reflects the number of care opportunities fulfilled at each hospital (numerator) divided by the number of opportunities to provide care (denominator). Excluded from both numerator and denominator were particular interventions that were contra-indicated, not applicable, not indicated in, or declined by, individual patients.

**Supplementary Table 4: Quality Indicators for STEMI patients according to socioeconomic status, stratified by presence of diabetes mellitus (DM) (ESC ACVC and OBQI)**

| **Variables** | **Quintile 1 (most deprived)** | | **Quintile 2** | | **Quintile 3** | | **Quintile 4** | | **Quintile 5 (most affluent) (** | |
| --- | --- | --- | --- | --- | --- | --- | --- | --- | --- | --- |
|  | Diabetes Mellitus (DM) **(n= 10,841)** | No DM **(n= 50,370)** | DM **(n= 10,257)** | No DM **(n= 51,082)** | DM **(n= 8,660)** | No DM **(n= 48,970)** | DM **(n= 6,827)** | No DM **(n= 43,395)** | DM **n= 5,917)** | No DM **(n= 41,232)** |
| **Reperfusion within 12 h of presentation** | 6,990/7,138 (98) | 36,380/36,827 (99) | 6,744/7,138 (98) | 37,236/37,736 (99) | 5,739/5,890 (97) | 36,004/36,491 (99) | 4,473/4,563 (99) | 31,676/32,121 (99) | 3,913/3,985 (99) | 30,409/30,829 (99) |
| **Door-to-balloon time <60 min** | 4,719/7,138 (66) | 26,924/36,827 (73) | 4,806/6,910 (70) | 28,315/37,736 (75) | 4,207/5,890 (71) | 27,718/36,491 (76) | 3,240/4,563 (71) | 24,459/32,121 (76) | 2,787/3,985 (70) | 23,601/30,829 (77) |
| **Door-to-balloon time <120 min** | 6,310/7,138 (88) | 33,823/36,827 (92) | 6,161/6,910 (89) | 34,833/37,736 (92) | 5,293/5,890 (90) | 33,727/36,491 (92) | 4,112/4,563 (90) | 29,702/32,121 (92) | 3,586/3,985 (90) | 28,577/30,829 (93) |
| **Revascularization (PCI/CABG)** | 7,147/10,732 (67) | 33,751/49,682 (68) | 6,795/10,120 (67) | 34,588/50,306 (69) | 5,649/8,537 (66) | 32,978/48,252 (68) | 4,395/6,738 (65) | 29,176/42,736 (68) | 3,876/5,836 (66) | 28,266/40,585 (70) |
| **Left ventricular ejection fraction assessed** | 5,772.7,772 (74) | 26,454/35,609 (74) | 5,674/7,610 (75) | 26,933/36,592 (73) | 4,601/6,299 (73) | 25,355/35,195 (72) | 3,721/5,048 (74) | 22,684/31,697 (72) | 3,229/4,411 (73) | 21,263/29,969 (71) |
| **DAPT received on discharge** | 8,989/10,442 (86) | 41,551/48,343 (86) | 8,528/9,922 (86) | 42,833/49,483 (87) | 7,278/8,385 (87) | 41,199/47,257 (87) | 5,731/6,617 (87) | 36,785/41,977 (88) | 4,983/5,750 (87) | 35,122/40,032 (88) |
| **ACE inhibitor or ARB on discharge for those with moderate and severe LVSD (%)** | 2,828/3,399 (83) | 11,989/13,915 (86) | 2,746/3,319 (83) | 11,975/13,947 (86) | 2,263/2,703 (84) | 11,323/13,272 (85) | 1,822/2,161 (84) | 10,051/11,720 (86) | 1,576/1,858 (85) | 9,449/11,023 (86) |
| **Mean OBQI score** | 89.0 | 88.9 | 87.6 | 88.5 | 88.4 | 88.6 | 88.0 | 88.9 | 88.4 | 88.7 |
| **Cardiac rehabilitation (%)** | 8,422/9,965 (85) | 41,226/46,703 (88) | 7,815/9,457 (83) | 41,490/47,349 (88) | 6,681/7,966 (84) | 39,908/45,358 (88) | 5,269/6,309 (84) | 35,631/40,322 (88)_ | 4,561/5,488 (85) | 33,806/38,371 (88) |

Data are expressed as proportions (%) unless indicated otherwise. Denominators represent the total number of participants with a data point collected; numerators represent the number of those participants for whom the variable of interest was present

Opportunity-based care score. The score consisted of six evidence-based processes of care: prescription of aspirin, a thienopyridine inhibitor, a β-blocker, an ACE inhibitor and a hydroxymethylglutaryl-coenxyme A (HMG CoA) reductase enzyme inhibitor (statin) and enrolment onto a cardiac rehabilitation programme at the time of discharge. The score reflects the number of care opportunities fulfilled at each hospital (numerator) divided by the number of opportunities to provide care (denominator). Interventions that were contraindicated, not applicable or not indicated in or declined by individual participants were excluded from both the numerator and the denominator

**Supplementary Table 5: Survival analysis for patients with diabetes mellitus with AMI according to socioeconomic status from propensity score matched population**

| **Outcome variables** | **Adjusted hazard ratio for patients compared to most affluent Quintile (Q5) according to IMD Score (95% CIs) (n=87,485)** | | | | | | | |
| --- | --- | --- | --- | --- | --- | --- | --- | --- |
|  | **Quintile 1 (most deprived)** | **P-value** | **Quintile 2** | **P-value** | **Quintile 3** | **P-value** | **Quintile 4** | **P-value** |
| **Primary Outcomes** | | |  |  |  |  |  |  |
| **Thirty-day mortality** | 0.97 (0.90-1.06) | 0.541 | 1.02 (0.94-1.11) | 0.651 | 1.00 (0.92-1.08) | 0.993 | 0.99 (0.91-1.08) | 0.878 |
| **One-year mortality** | 1.05 (0.99-1.11) | 0.083 | 1.09 (1.03-1.15) | 0.001 | 1.05 (1.00-1.11) | 0.064 | 1.02 (0.97-1.08) | 0.431 |
| **Five-year mortality** | 1.14 (1.10-1.18) | <0.001 | 1.12 (1.08-1.16) | <0.001 | 1.06 (1.02-1.10) | 0.001 | 1.02 (0.98-1.06) | 0.295 |
| **Overall mortality** | 1.15 (1.11-1.19) | <0.001 | 1.12 (1.08-1.15) | <0.001 | 1.06 (1.03-1.10) | <0.001 | 1.03 (0.99-1.06) | 0.137 |

Adjusted Hazard ratios are presented with 95% CIs, adjusted for: age, sex, year, hospital region, heart rate, blood pressure, co-morbid conditions (hypertension, history of asthma or COPD, history of CVA or PVD, hypercholesterolaemia, family history of coronary artery disease, smoking history, previous AMI, previous PCI and previous CABG ), cardiac arrest, LV systolic function, Killip classification and ischaemic ECG changes, medication strategy (warfarin, LMWH, UFH, glycoprotein B3A, aspirin, P2Y12 inhibitor, statin, beta-blockers and ACE inhibitors), invasive angiography while an inpatient, and revascularisation by PCI or CABG.

Applied to a one-to-one propensity score matched population (n=87,485) with DM and without, matched for the above covariates.

**Supplementary Table 6: Survival analysis for patients without diabetes mellitus with AMI according to socioeconomic status from propensity score matched population**

| **Outcome variables** | **Adjusted hazard ratio for patients compared to most affluent Quintile (Q5) according to IMD Score (95% CIs) (n=87,485)** | | | | | | | |
| --- | --- | --- | --- | --- | --- | --- | --- | --- |
|  | **Quintile 1 (most deprived)** | **P-value** | **Quintile 2** | **P-value** | **Quintile 3** | **P-value** | **Quintile 4** | **P-value** |
| **Primary Outcomes** | | |  |  |  |  |  |  |
| **Thirty-day mortality** | 1.05 (0.96-1.14) | 0.262 | 0.99 (0.91-1.07) | 0.749 | 0.95 (0.88-1.03) | 0.213 | 0.90 (0.82-0.98) | 0.013 |
| **One-year mortality** | 1.10 (1.04-1.17) | 0.001 | 1.01 (0.96-1.07) | 0.685 | 0.97 (0.92-1.03) | 0.346 | 0.91 (0.86-0.97) | 0.003 |
| **Five-year mortality** | 1.18 (1.14-1.23) | <0.001 | 1.06 (1.02-1.10) | 0.004 | 1.02 (0.98-1.06) | 0.240 | 0.97 (0.93-1.01) | 0.158 |
| **Overall mortality** | 1.21 (1.17-1.26) | <0.001 | 1.09 (1.05-1.12) | <0.001 | 1.04 (1.01-1.12) | 0.015 | 0.98 (0.95-1.02) | 0.277 |

Adjusted Hazard ratios are presented with 95% CIs, adjusted for: age, sex, year, hospital region, heart rate, blood pressure, co-morbid conditions (hypertension, history of asthma or COPD, history of CVA or PVD, hypercholesterolaemia, family history of coronary artery disease, smoking history, previous AMI, previous PCI and previous CABG ), cardiac arrest, LV systolic function, Killip classification and ischaemic ECG changes, medication strategy (warfarin, LMWH, UFH, glycoprotein B3A, aspirin, P2Y12 inhibitor, statin, beta-blockers and ACE inhibitors), invasive angiography while an inpatient, and revascularisation by PCI or CABG.

Applied to a one-to-one propensity score matched population (n=87,485) with DM and without, matched for the above covariates.

**Supplementary Table 7: Adjusted probability of one-year mortality according to interaction of one-year mortality with IMD Quintile and diabetes mellitus status from propensity score matched population**

| **Outcome variables** | **Adjusted probability of one-year mortality for patients according to interaction between socioeconomic status and diabetes mellitus status (95% CIs)** | |
| --- | --- | --- |
| **Combinations of IMD Quintile and DM status** | **Adjusted one-year mortality with 95% CIs (%)** | **P-value for interaction** |
| **Quintile 1 (most deprived) and no-DM** | 17.7 (17.2-18.2) | <0.001 |
| **Quintile 1 (most deprived) and DM** | 20.2 (19.8-20.7) |  |
| **Quintile 2 and no-DM** | 16.8 (16.4-17.2) | <0.001 |
| **Quintile 2 and DM** | 20.8 (20.4-21.3) |  |
| **Quintile 3 and no-DM** | 16.1 (15.7-16.5) | <0.001 |
| **Quintile 3 and DM** | 20.0 (19.6-20.5) |  |
| **Quintile 4 and no-DM** | 15.6 (15.1-16.1) | <0.001 |
| **Quintile 4 and DM** | 19.8 (19.2-20.3) |  |
| **Quintile 5 (most affluent) and no-DM** | 16.2 (15.7-16.7) | <0.001 |
| **Quintile 5 (most affluent) and DM** | 19.2 (18.7-19.8) |  |

**P-values displayed are for interaction comparison within Quintile group according to presence of diabetes mellitus (DM). Model applied to one-one matched population following propensity score matching.** Adjusted one-year mortality presented with 95% CIs, adjusted for: age, sex, year, hospital region, heart rate, blood pressure, co-morbid conditions (hypertension, history of asthma or COPD, history of CVA or PVD, hypercholesterolaemia, family history of coronary artery disease, smoking history, previous AMI, previous PCI and previous CABG ), cardiac arrest, LV systolic function, Killip classification and ischaemic ECG changes, medication strategy (warfarin, LMWH, UFH, glycoprotein B3A, aspirin, P2Y12 inhibitor, statin, beta-blockers and ACE inhibitors), invasive angiography while an inpatient, and revascularisation by PCI or CABG.

Applied to a one-to-one propensity score matched population (n=87,485) with DM and without, matched for the above covariates.

**Supplementary Table 8: Survival analysis for patients with diabetes mellitus with AMI according to socioeconomic status**

| **Outcome variables** | **Adjusted hazard ratio for patients compared to most affluent Quintile (Q5) according to IMD Score (95% CIs) (n=87,485)** | | | | | | | |
| --- | --- | --- | --- | --- | --- | --- | --- | --- |
|  | **Quintile 1 (most deprived)** | **P-value** | **Quintile 2** | **P-value** | **Quintile 3** | **P-value** | **Quintile 4** | **P-value** |
| **Primary Outcomes** | | |  |  |  |  |  |  |
| **Thirty-day mortality** | 1.00 (0.94-1.06) | 0.937 | 1.07 (1.01-1.13) | 0.030 | 1.02 (0.96-1.09) | 0.507 | 1.04 (0.98-1.11) | 0.202 |
| **One-year mortality** | 1.05 (0.01-1.09) | 0.021 | 1.09 (1.05-1.13) | <0.001 | 1.04 (1.00-1.08) | 0.051 | 1.03 (0.99-1.07) | 0.136 |
| **Five-year mortality** | 1.11 (1.09-1.15) | <0.001 | 1.11 (1.08-1.14) | <0.001 | 1.07 (1.04-1.10) | <0.001 | 1.03 (1.00-1.06) | 0.057 |
| **Overall mortality** | 1.12 (1.10-1.15) | <0.001 | 1.10 (1.08-1.13) | <0.001 | 1.07 (1.04-1.09) | <0.001 | 1.03 (1.00-1.05) | 0.024 |

Adjusted Hazard ratios are presented with 95% CIs, adjusted for: age, sex, year, hospital region, heart rate, blood pressure, co-morbid conditions (hypertension, obesity, history of asthma or COPD, history of CVA or PVD, hypercholesterolaemia, family history of coronary artery disease, smoking history, previous AMI, previous PCI and previous CABG ), cardiac arrest, LV systolic function, Killip classification and ischaemic ECG changes, medication strategy (warfarin, LMWH, UFH, glycoprotein B3A, aspirin, P2Y12 inhibitor, statin, beta-blockers and ACE inhibitors), admission to cardiology ward, invasive angiography while an inpatient, and revascularisation by PCI or CABG.

**Supplementary Table 9: Survival analysis for patients without diabetes mellitus with AMI according to socioeconomic status**

| **Outcome variables** | **Adjusted hazard ratio for patients compared to most affluent Quintile (Q5) according to IMD Score (95% CIs) (n=87,485)** | | | | | | | |
| --- | --- | --- | --- | --- | --- | --- | --- | --- |
|  | **Quintile 1 (most deprived)** | **P-value** | **Quintile 2** | **P-value** | **Quintile 3** | **P-value** | **Quintile 4** | **P-value** |
| **Primary Outcomes** | | |  |  |  |  |  |  |
| **Thirty-day mortality** | 1.06 (1.02-1.10) | 0.004 | 1.03 (1.00-1.07) | 0.079 | 0.99 (0.95-1.02) | 0.523 | 0.99 (0.95-1.02) | 0.434 |
| **One-year mortality** | 1.12 (1.09-1.15) | <0.001 | 1.07 (1.04-1.09) | <0.001 | 1.00 (0.98-1.03) | 0.726 | 1.00 (0.97-1.02) | 0.856 |
| **Five-year mortality** | 1.18 (1.16-1.20) | <0.001 | 1.11 (1.09-1.12) | <0.001 | 1.04 (1.02-1.06) | <0.001 | 1.02 (1.00-1.03) | 0.050 |
| **Overall mortality** | 1.21 (1.20-1.23) | <0.001 | 1.12 (1.11-1.14) | <0.001 | 1.06 (1.04-1.07) | <0.001 | 1.02 (1.01-1.04) | 0.002 |

Adjusted Hazard ratios are presented with 95% CIs, adjusted for: age, sex, year, hospital region, heart rate, blood pressure, co-morbid conditions (hypertension, obesity, history of asthma or COPD, history of CVA or PVD, hypercholesterolaemia, family history of coronary artery disease, smoking history, previous AMI, previous PCI and previous CABG ), cardiac arrest, LV systolic function, Killip classification and ischaemic ECG changes, medication strategy (warfarin, LMWH, UFH, glycoprotein B3A, aspirin, P2Y12 inhibitor, statin, beta-blockers and ACE inhibitors), admission to cardiology ward, invasive angiography while an inpatient, and revascularisation by PCI or CABG.

**Supplementary Figure 1**


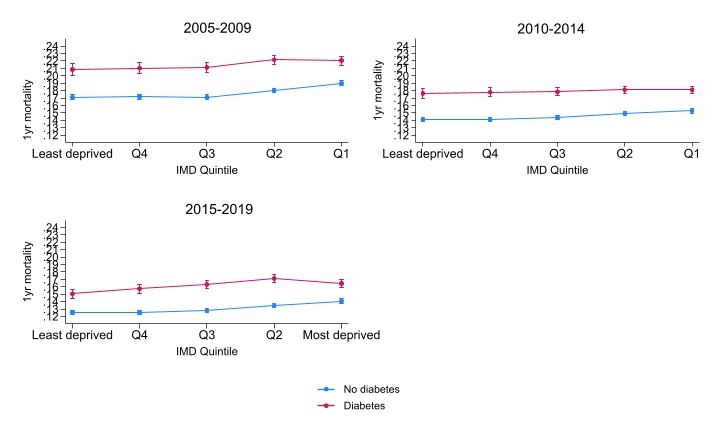


Adjusted one year mortality calculated from logistic regression model, adjusting for age, sex, ethnicity (classified according to the MINAP categories; White, Black, Asian, Mixed and Other), year of admission, hospital region, admission heart rate, admission systolic blood pressure, co-morbid conditions (hypertension, history of asthma or chronic obstructive pulmonary disease (COPD), history of cerebrovascular accident (CVA) or peripheral vascular disease (PVD), hypercholesterolaemia, family history of coronary artery disease, smoking history, previous AMI, history of angina, history of previous PCI and previous CABG ), medication strategy (aspirin, P2Y12 inhibitor, LMWH, fondaparinux, warfarin, unfractionated heparin (UFH), glycoprotein B3A, ACE inhibitor/ARB, statin, beta-blocker), cardiac arrest, left ventricular (LV) systolic function, Killip classification and ischaemic ECG changes, and invasive coronary angiography, revascularisation by percutaneous coronary intervention (PCI) or coronary artery bypass grafts surgery (CABG).
